# Topological Design of a Lightweight Sandwich Aircraft Spoiler

**DOI:** 10.3390/ma12193225

**Published:** 2019-10-01

**Authors:** Jie Liu, Haifeng Ou, Junfeng He, Guilin Wen

**Affiliations:** Center for Research on Leading Technology of Special Equipment, School of Mechanical and Electric Engineering, Guangzhou University, Guangzhou 510006, China; jliu@gzhu.edu.cn (J.L.); 2111707002@e.gzhu.edu.cn (H.O.); 1407200094@e.gzhu.edu.cn (J.H.)

**Keywords:** aircraft spoiler, topology optimization, lattice structure, high stiffness-to-weight ratio

## Abstract

In this study, a lightweight sandwich aircraft spoiler (AS) with a high stiffness-to-weight ratio was designed. Excellent mechanical properties were achieved by the synthetic use of topology optimization (TO), lattice structure techniques, and high-performance materials, i.e., titanium alloy and aluminum alloy. TO was first utilized to optimize the traditional aircraft spoiler to search for the stiffest structure with a limited material volume, where titanium alloy and aluminum alloy were used for key joints and other parts of the AS, respectively. We then empirically replaced the fine features inside the optimized AS with 3D kagome lattices to support the shell, resulting in a lightweight sandwich AS. Numerical simulations were conducted to show that the designed sandwich AS exhibited good mechanical properties, e.g., high bending rigidity, with a reduction in weight by approximately 80% when compared with that of the initial design model. Finally, we fabricated the designed model with photosensitive resin using a 3D printing technique.

## 1. Introduction

A spoiler in aircrafts is a piece of equipment aimed at intentionally reducing the lift component of an airfoil in a controlled way [1]. Considering the demanding requirements for a high stiffness-to-weight ratio in the aerospace industry, a spoiler should be designed to be lightweight and with a high stiffness [2]. Traditionally, they have been designed using a trial-and-error method, which is always time-consuming and normally too conservative. Recently developed topology optimization (TO) methods [3,4,5] can overcome these drawbacks.

TO methods, which serve as efficient tools, can produce various novel candidates for engineering structures. Initially, these methods purely play a role in the conceptual design process in industry, following shape optimization, size optimization, and numerical verifications. The main reason behind this is the gap between the complexity and intricacy of an optimum solution and traditional manufacturing techniques, although various manufacturing constraints are considered [6]. This gap has been significantly overcome with the rapid development of additive manufacturing (AM), e.g., 3D printing [7]. Although showing a promising perspective, with the ability of reducing geometric intricacy restrictions imposed on topology optimization by conventional manufacturing, several key problems must be dealt with for AM, e.g., the support structure design [8,9,10]. The superiority of AM may be more prominent when one attempts to design and fabricate sandwich structures. Sandwich structures, constructed by attaching two thin (yet stiff) skins to a lightweight (but thick) core, are typically lightweight structures with high stiffness. Because of the use of the core, the whole structure can exhibit high mechanical performance using normally low-strength material. Various cores have been proposed, e.g., tetrahedron [11], 3D kagome [12], pyramid [13], honeycomb [14], origami [15], etc. Notice that the advantages and drawbacks of these cores are not covered in this work and readers are referred to review papers [16,17] and references therein. In this study, 3D kagome is employed as the core to construct the sandwich aircraft spoiler (AS). 

Using TO methods, several components of the aircraft have been designed, mainly including airframe structures and stiffener ribs for aircraft panels [2]. Using a bi-level optimization scheme, two Airbus pylons were designed by combing TO methods and geometric optimization [18]. Zhu et al. [19] proposed a novel TO method to improve the stiffness and strength of an aircraft skin stretch-forming die. A Heaviside-function-based directional growth topology parameterization has been developed to achieve stiffener layout designs [20], having the potential to design stiffener ribs for aircraft panels. Krog et al. designed aircraft wing box ribs by using TO methods [21]. By tackling coupled fluid–structure problems, morphing aircraft structures were designed by using a multidisciplinary TO method [22]. In addition, multi-component design problems of aircrafts have been extensively studied by Zhang and his co-workers [23,24,25]. However, these final optimized designs [26,27,28] are generally not able to be fabricated directly since these designs (1) have no direct link with CAD modelling system and (2) their edges normally need to be smoothed. Recently proposed morphable moving component/void (MMC/MMV) [29,30,31] methods have the ability to seamlessly integrate topology optimization in CAD modeling systems, which can overcome problem (1). For problem (2), numerous methods, including employing higher-order finite elements or refined meshes [32,33,34], mesh adaptive strategies [35,36], and high-resolution techniques [37,38] have been proposed. Note that level-set methods can inherently produce structures with smooth edges [39,40]. 

Alternatively, commercial software, e.g., Abaqus [41], embedded TO algorithm can also produce optimized structures with clear boundaries. In this work, we employ Abaqus to optimize the AS with clear boundary features and utilize Solidworks [42] to remodel a CAD model of the optimized AS. The internal fine structures established in Solidworks, which are very complex, are manually replaced by 3D kagome cores to improve the manufacturability and the stiffness-to-weight ratio. In addition, to further reduce the weight and improve the strength of AS, aluminum alloy and titanium alloy are synthetically applied to the key joints and the other parts, respectively. We conduct numerical simulations to investigate whether the designed aircraft spoiler can meet the service environment. Finally, a novel aircraft spoiler model is fabricated by using photosensitive resin. 

## 2. Materials and Methods

### 2.1. Topology Optimization Method 

TO aims to obtain the optimal material layout under the prescribed loading and boundary conditions with the given material. Density-based methods treat the material density of each element as the design variable between 0 (void element) to 1 (solid element) by allowing the material to take intermediate densities (gray element). The Young’s modulus of the elements in the design domain is parameterized with design variable and the properties of intermediate densities are artificially penalized. To achieve penalized intermediate densities, the Young’s modulus of the *e*th element can be expressed as [3],

(1) $$E_{e}=x_{e}^{p}E_{s}$$

where *E_s_* is the Young’s modulus of the solid element, *x_e_* is the relative density of the material element (i.e., the design vector of the element densities), and *p* as the penalization power is used to suppress intermediate density and ensures good convergence to 0/1 designs, which usually has a value of 3. 

Let the structure be discretized into *N* finite elements (i.e., the number of design variables) and let ***k****_e_* be the element stiffness matrix, then, the global stiffness matrix can be expressed as:

(2) $$K=\sum_{e=1}^{N}x_{e}^{p}k_{e}.$$

To maximize the structural stiffness is equivalent to minimize the structural compliance, thus the TO problem can be formulated as [2,3,4],

(3) $$\begin{array}{cc} \mathrm{Min}: & c(x)=U^{T}KU=\sum_{e=1}^{N}{\left(x_{e}\right)}^{p}u_{e}^{T}k_{e}u_{e}\\ s.t.: & \frac{V(x)}{V_{0}}=f\\ : & KU=F\\ : & 0<x_{\min}\leq x\leq 1 \end{array}$$

where *c*(***x***) is the objective function, ***F*** is the global load vector, ***U*** and ***u****_e_* are the global and element displacement vectors, respectively, ***V***_(*x*)_ and ***V***_0_ are the solid material volume and design domain volume, respectively, *f* is the prescribed volume fraction (i.e., the ratio of optimized structure to design domain), and ***x***_min_ is a vector of minimum relative densities, which avoids the non-positive definite stiffness matrix, and its value is usually set to be 0.001.

The optimization presented in Equation (3) can be solved by utilizing many methods, e.g., the method of moving asymptotes (MMA) [43], optimality criterion (OC) method [44], linear or sequential quadratic programming methods [45,46], the ESO method [47,48], etc. In this study, Solid Isotropic Material with Penalty (SIMP)and OC methods were used to optimize the AS, in which OC is the solver.

### 2.2. Design Problem Definition

**Figure 1:**
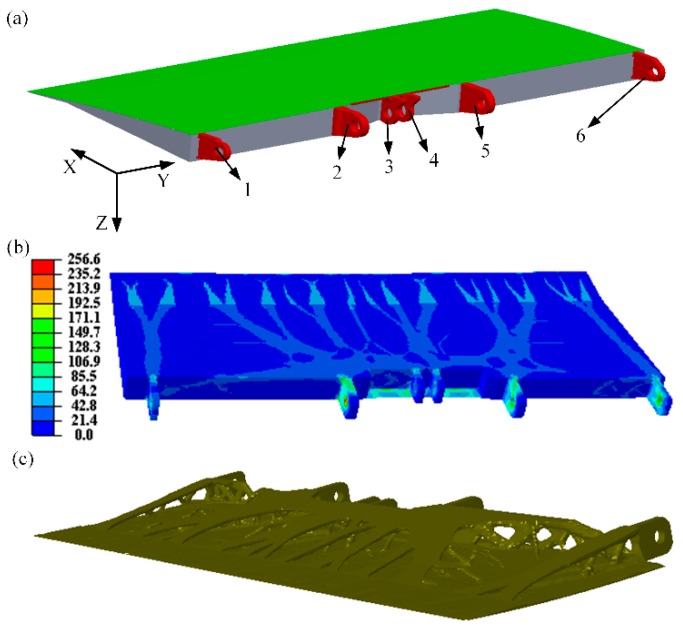
(**a**) An initial aircraft spoiler (AS) design domain; (**b**) the optimized AS and von Mises stress distributions; (**c**) remodeled AS.

The initial design domain of AS is shown in Figure 1a, which consists of a skin (highlighted in green), joints (highlighted in red), and the main structure (highlighted in gray). The initial thickness of the skin was set to be 1 mm. The skin domain had a length and width of 1220 × 426 mm, and was discretized by 21,551 S4 elements. The remaining domain had a length of 1220 mm and a width of 470 mm and is occupied by 149,779 C3D8 elements. Two non-design domains, the skin and joints, are defined to maintain the integrity of the final design. The objective function is to minimize the structural flexibility under a volume constraint of 20%. The *x*-direction of all joints is constrained, *y*-direction of joints 1 and 2 is constrained, and *z*-direction of joints 2, 3, 4, and 5 is fixed. A distributed uniform load of 20,000 Pa is applied at the structural upper skin along the vertical skin. Joints and the skin were constructed using titanium alloy and aluminum alloy was used to form the other parts, of which the properties are shown in Table 1. The volume and the weight of the initial AS were 14.1 × 10^−3^ mm^3^ and 40.395 kg, respectively. It should be noted that all the initial design parameters, including geometric and material parameters, in this study were provided by an aircraft design and research institute in China. In addition, although the tensile and compressive strength of aluminum are very close, the values we used in this study exhibiting apparent difference are provided by the institute mentioned above.

## 3. Results and Discussions

**Figure 2:**
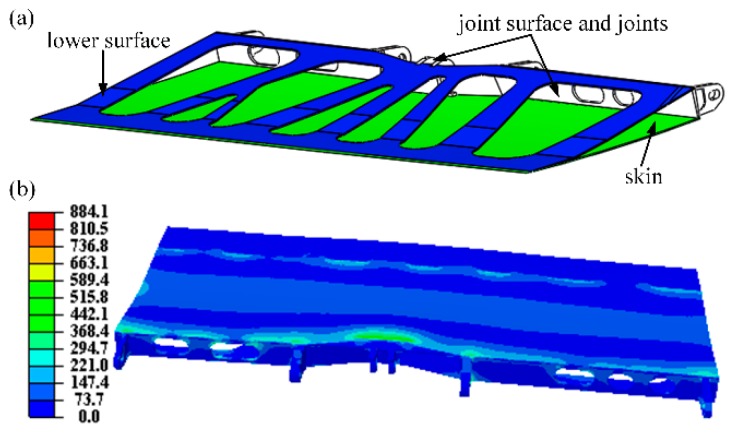
(**a**) The remodeled AS excluding the inner structures and (**b**) its von Mises stress distribution.

Figure 1b shows the optimal material layout of AS and the von Mises stress distribution. The maximum stress (stress concentration) of this structure occurs at the joints, as expected, with a maximum value of 256.6 MPa. To remodel the optimized AS, the regions with material density greater than 0.4 are replaced with solid materials, otherwise removed (Figure 1c). The inner fine features are removed to increase the manufacturability (Figure 2a). The remodeled AS includes the skin (colored green), the lower surface (colored blue), and joints (colored gray). In addition, sharp corners are rounded to avoid the stress concentration. The internal structure, linking the skin and the lower surface, is removed to make room for the lattice structure. The material for the lattice structure is aluminum, whose properties are presented in Table 1. Note that the maximum stress occurs at the surface of the joints. Hence, we increased the thickness of the joint to 4 mm. Moreover, to ensure the designed structure can be easily fabricated by using a normal 3D printing machine, the thickness of both the skin and lower surface is set to be 2.5 mm. 

**Figure 3:**
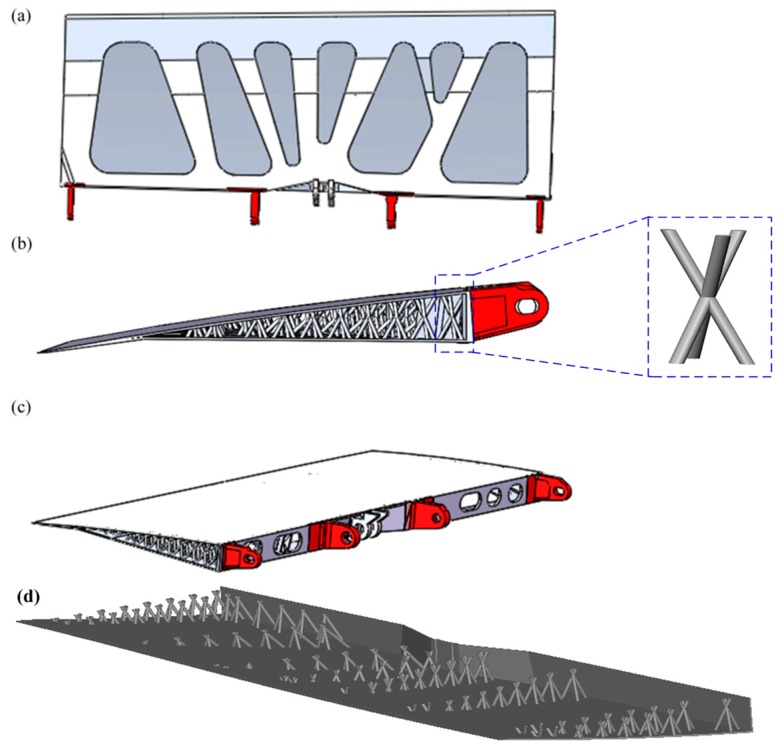
The designed aircraft spoiler: (**a**) top view, (**b**) lateral view, and (**c**) isometric view, (**d**) the view showing the lattice structure.

Figure 2b presents the corresponding stress distribution of the remodeled AS without the inside fine structures. The maximum stress becomes 884.1 MPa, which is easy to understand since the internal support was removed. Subsequently, a 3D kagome lattice structure was added to the inside of the remodeled AS. The rod diameter of each lattice unit was set to 5 mm. The height of the 3D kagome lattice structures was determined adaptively, based on the distance between the skin and the lower surface. Note that the positions of the lattice structures are carefully selected according to the stress distribution presented in Figure 2b; in other words, we employ the 3D kagome lattice structure to support the skins of the spoiler to reduce the stress concentration. It should be underlined that the detailed process to determine the specific lattice geometry is as follows. The basic unit cells of the 3D kagome lattice [12] close to the joints are first designed in Solidworks (see Figure 3b) according to the geometry of the optimized structure presented in Figure 2a. Next, we distributed the unit cell longitudinally along the AS by carefully considering the geometric changes of the lower surface of the AS. Then, we used the upper surface of the AS to cut the lattice structures and remove the redundant parts. Finally, we achieced the specific lattice, as shown in Figure 3d. It should be noted that experience is needed in this step. However, structural optimization techniques [49] can be further adopted to determine the geometry and the position of the unit cell, which is out of the scope of this study.

Figure 3a–c show the top view, the lateral view, and the isometric view of the final designed AS, respectively. The stress concentration of the skin or the lower surface should be significantly reduced by adding lattice structures. However, the joints, which are non-designable, can be regarded as the support end of one cantilever and may exceed the maximum stress since we have added more materials to the skin or the lower surface by increasing their thicknesses. Therefore, titanium alloy instead of the aluminum alloy is used to construct the key joints, i.e., joints 1, 2, 5, 6. Aluminum alloy is used for the other parts to further reduce the whole weight. 

**Figure 4:**
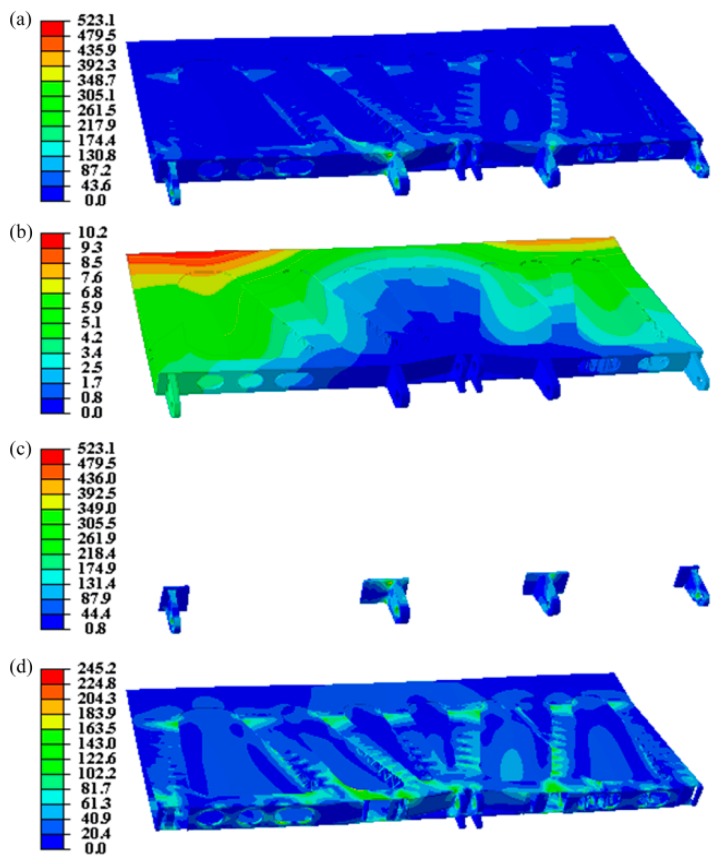
Numerical results for the final designed AS: (**a**) von Mises stress distribution; (**b**) displacement; (**c**) von Mises stress distribution of key joints 1, 2, 5, 6 and adjacent regions; (**d**) von Mises stress distribution except for joints 1, 2, 5, 6 and adjacent regions.

**Figure 5:**
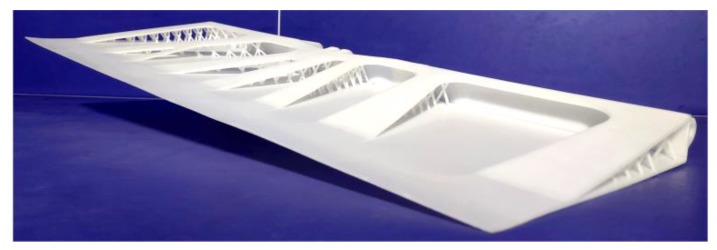
The real model for the designed AS.

Figure 4a,b present the von Mises stress distribution and displacement distribution of the final design, showing that the maximum stress and the maximum displacement are 523.1 MPa and 10.19 mm, respectively. The small displacement means high stiffness of the structure. In addition, the corresponding stress distributions of the regions with different material properties are also depicted in Figure 4c,d. Specifically, the maximum stress of the joints 1, 2, 5, 6 and the adjacent regions is 523.1 MPa (Figure 4c) and for other regions is 245.2 MPa. These two values are both smaller than the yield stress of the corresponding material used. The volume of the designed aircraft spoiler is 2.63 × 10^−3^ mm^3^, and the weight is 7.772 kg. Moreover, compared with the initial design structure, the volume and weight of the final designed AS are reduced by 81.35% and 80.76%, respectively. Therefore, we have designed a lightweight sandwich AS with a high stiffness-to-weight ratio by using titanium alloy and aluminum alloy. Finally, considering the cost of 3D printing for metals, we fabricated the final designed AS with photosensitive resin by using a Jinshi high speed light curing 3D printer (Type: JS7255) to show its real configuration, as shown in Figure 5.

It should be stressed that the designed sandwich AS is indeed not the best one since empirical method is used and TO is normally unable to obtain the global optimum. Alternatively, TO for lattice structures [49] may provide guidance in the design of AS. However, considering that it is a large-scale problem and the design domain is geometrically complex, it is not an easy task [50]. Moreover, this study purely aims at showing a promising way to design a novel AS with a high stiffness-to-weight ratio. However, for real aircraft spoilers, multiple loads condition and buckling constraints should be carefully considered.

## 4. Conclusions

We have designed a sandwich AS with a high stiffness-to-weight ratio by using two materials, i.e. titanium alloy and aluminum alloy. TO are used to search for the best material distribution with maximizing the structural stiffness as the objective. The internal support materials obtained from the TO results are removed and subsequently replaced by 3D kagome lattice structures. To further improve the strength and at the same time reduce the weight of the aircraft spoiler, joints 1, 2, 5, 6, the key bearing components, use titanium alloy, while the other parts utilize aluminum alloy. Results show that the volume and weight of the designed aircraft spoiler are reduced by 81.35% and 80.76%, respectively, when compared with those of the initial design structure. In addition, the maximum stress and the maximum displacement of the final designed AS are 523.1 MPa and 10.19 mm, respectively, demonstrating that it can meet its service environment. We finally fabricated a real model for the novel sandwich AS by using 3D printing. It is worth highlighting that it is not an easy task to weld titanium with aluminum, and further studies are needed [51]. Moreover, it is highly necessary to conduct experiments to verify the mechanical performances of the optimized structure. However, it is very hard to achieve this goal, and the authors may explore this field in the future.

## Figures and Tables

**Table 1:** Properties of the used materials.

Material	Titanium Alloy	Aluminum Alloy
Density (kg/m^3^)	4500	2760
Tensile strength (MPa)	900	450
Compressive strength (MPa)	880	270
Modulus of elasticity (GPa)	108	68
Poisson’s ratio	0.33	0.33
